# CPNE1 is a target of miR-335-5p and plays an important role in the pathogenesis of non-small cell lung cancer

**DOI:** 10.1186/s13046-018-0811-6

**Published:** 2018-07-03

**Authors:** Haicheng Tang, Jianjie Zhu, Wenwen Du, Shunlin Liu, Yuanyuan Zeng, Zongli Ding, Yang Zhang, Xueting Wang, Zeyi Liu, Jianan Huang

**Affiliations:** 1 Department of Respiratory Medicine, the First Affiliated Hospital of Soochow University, Suzhou, 215006 China; 2 Suzhou Key Laboratory for Respiratory Diseases, Suzhou, 215006 China; 3 Institute of Respiratory Diseases, Soochow University, Suzhou, 215006 China; 4 Department of Respiratory Medicine, The First People’s Hospital of Yancheng City, Yancheng, 224001 China

**Keywords:** Non-small cell lung cancer (NSCLC), Copine 1 (CPNE1), microRNA, miR-335-5p

## Abstract

**Background:**

Despite advances in diagnosis and treatment, the survival of non-small cell lung cancer (NSCLC) patients remains poor. There is therefore a strong need to identify potential molecular targets for the treatment of NSCLC. In the present study, we investigated the function of CPNE1 in the regulation of cell growth, migration and invasion.

**Methods:**

Quantitative real-time PCR (qRT-PCR) was used to detect the expression of *CPNE1* and miR-335-5p. Western blot and immunohistochemical assays were used to investigate the levels of CPNE1 and other proteins. Flow cytometry was used to determine cell cycle stage and apoptosis. CCK-8 and clonogenic assays were used to investigate cell proliferation. Wound healing, migration and invasion assays were used to investigate the motility of cells. A lung carcinoma xenograft mouse model was used to investigate the in vivo effects of CPNE1 overexpression.

**Results:**

We observed that knockdown of CPNE1 and increased expression of miR-335-5p inhibits cell proliferation and motility in NSCLC cells, and found that CPNE1 was a target of miR-335-5p. In addition, our data indicated that CPNE1 inhibition could improve the clinical effects of EGFR-tyrosine kinase inhibitors.

**Conclusions:**

The present results indicate that CPNE1 may be a promising molecular target in the treatment of NSCLC.

**Electronic supplementary material:**

The online version of this article (10.1186/s13046-018-0811-6) contains supplementary material, which is available to authorized users.

## Background

Lung cancer is the leading cause of cancer-related death in China and worldwide [1, 2]. Non-small cell lung cancer (NSCLC) accounts for 85% of lung cancers. Despite the advances in cancer research and treatment, the prognosis of NSCLC is still poor, with the 5-year survival rate being only 15% [3]. Although new drugs such as gefitinib and erlotinib have been shown to be beneficial, especially in patients with the target mutations, the survival and outcome have not changed dramatically. Therefore, it is important to understand the pathogenesis of NSCLC and identify new treatment targets.

Copines are a family of calcium-dependent phospholipid-binding proteins that are evolutionally conserved in various eukaryotic organisms and protists [4]. Currently, nine family members of the copine have been identified [5, 6]. It has been reported that Copine1, which is encoded by CPNE1, includes two tandem C2 domains at the N-terminus and an A domain at the C-terminus [4]. The C2 domains act as calcium-dependent phospholipid-binding motifs and may be involved in cell signaling and/or membrane trafficking pathways [7]. The A domain is named after von Willebrand Factor, a plasma and extracellular matrix protein. It has been studied in integrins and several extracellular matrix proteins and appears to function as a protein-binding domain [8]. A recent study showed that CPNE3 is upregulated and can enhance cell metastasis in NSCLC [9]. Moreover, it has been shown that CPNE3 interacts with ErbB2 to promote tumor cell migration [10, 11]. Finally, a recent research has shown that the expression level of CPNE1 is higher in prostate cancer than in normal prostate tissue [12]. Based on all these findings, it would be interesting to investigate whether CPNE1 can interact with members of the ErbB family, especially EGFR. However, the expression level of CPNE1 in NSCLC is unknown.

EGFR is expressed more abundantly in malignant than in normal tissue, and in the case of NSCLC, EGFR expression is higher than that in normal tissue in 40–80% of the cases [13]. Moreover, overexpression of EGFR has been reported to be associated with poor prognosis [14]. This makes EGFR an important target for lung cancer therapy. It is known that EGFR-tyrosine kinase inhibitor (TKI) therapy can significantly improve the treatment outcomes of patients with lung cancers who harbor EGFR mutations; moreover, this therapy has also been found to benefit some lung cancer patients with wild-type EGFR [15, 16]. However, lung cancer cells inevitably acquire resistance to these inhibitors after 8−10 months [17–19]. This is probably because resistance to EGFR-TKI is mediated through multiple signaling pathways that converge upon cap-dependent translation in NSCLC cells expressing wild-type EGFR. Therefore, a better understanding of the molecular regulators of EGFR activation and its downstream signaling pathways could help with a more accurate selection of patients who are most likely respond to EGFR-targeted agents. Interestingly, our previous study showed that CD73 affected the efficacy of EGFR-targeted therapy in NSCLC cells with wild-type EGFR [20]. This finding is consistent with the results of a study on breast cancer [21]. There is a need to identify other molecular biomarkers that can predict the efficacy of EGFR-TKI therapy in patients with lung cancer with wild-type EGFR. In the present study, we examine the potential of CPNE1 as a potential marker.

miRNAs are a class of small noncoding RNAs that play essential roles in tumor development and progression via the regulation of various signaling networks that are associated with multiple cellular functions such as proliferation, migration, diagnosis and prognosis [22–25]. In particular, there is some evidence that miRNAs are closely related to the development of human lung cancer [20, 26, 27], and that comprehensive expression analysis of a large number of miRNAs could reflect the developmental lineage and differentiation state of human lung cancer [26]. In our previous study, we used miRNA arrays and found that miR-335-5p expression was significantly downregulated in NSCLC tissues [28]. To identify new targets of miR-335-5p that may play a role in NSCLC, in the present study, we predicted its target mRNAs using computational algorithms. Interestingly, miR-335-5p could bind to the 3′-UTR of CPNE1 mRNA. Further, we found that knockdown of CPNE1 inhibits and that increase in miR-335-5p expression promotes the cell cycle in NSCLC cells. Thus, miR-335-5p may be involved in lung cancer progression via the regulation of CPNE1. In addition, our data indicated that CPNE1 inhibition could improve the clinical effects of EGFR-TKIs. Taken together, the findings indicate that CPNE1 binds with EGFR and plays an important role in the tumorigenesis of NSCLC by targeting miR-335-5p.

## Methods

### Tissue samples

Paired NSCLC tissue and adjacent noncancerous lung tissue samples (60 of each) were collected with the informed consent of the patients from the First Affiliated Hospital of Soochow University between 2009 and 2013. The patients had been diagnosed with NSCLC based on their histological and pathological characteristics according to the Revised International System for Staging Lung Cancer. They had not undergone chemotherapy or radiotherapy prior to tissue sampling. The tissue samples were snap frozen and stored in a cryofreezer at − 80 °C. This study was approved by the Academic Advisory Board of Soochow University.

### Cell culture

Human NSCLC cell types A549, H1299, SPC-A1, HCC827, PC9, H1975 (lung adenocarcinoma cell lines), H226 (lung squamous carcinoma cell line) and BEAS-2B (human immortalized normal epithelial cell) were purchased from the Cell Bank of the Chinese Academy of Sciences (Shanghai, China). The cells were grown in RPMI 1640 medium containing 10% fetal bovine serum (FBS) (Gibco, Carlsbad, CA, USA) and l-glutamine (Invitrogen, Carlsbad, CA, USA) at 37 °C in a humidified atmosphere containing 5% CO_2_. Genetic characteristics of the cells were determined by Beijing Microread Genetics using the Goldeneye™ 20A Kit and ABI 3100. All cell lines were passaged for less than 3 months and tested in Jan 2016.

### Immunohistochemical assay

Immunohistochemical (IHC) analyses of tissues were conducted as described in our previous study [20]. In brief, the sections were incubated with Copine 1 antibody (diluted to 1:100; Abcam, 155,675) overnight at 4 °C, and then incubated with the corresponding biotinylated secondary antibodies. The reactions were developed using the DAB Kit (BD Bioscience, San Jose, CA, USA), and the sections were counterstained with hematoxylin.

### RNA interference

Two pre-designed short interfering RNA (siRNA) sequences, which target different coding regions of CPNE1, were directly synthesized (GenePharma). The target sequences of siRNA are as follows: siRNA-CPNE1–1: 5′-GGACUUCACUGGCUCCAAUTT-3′ (sense) and 5′-AUUGGAGCCAGUGAAGUCCTT-3′ (antisense); siRNA-CPNE1–2: 5′-GCAGGUCUC GCAUGAAUUUTT-3′ (sense) and 5′-AAAUUCAUGCGAGACCUGCTT-3′ (antisense). Scrambled siRNA was used as a negative control. Cells were transiently transfected with 100 pmol of siRNA sequences using Lipofectamine 2000 (Invitrogen). After 72 h of transfection, the cells were harvested for further experiments.

### RNA extraction and quantitative real-time PCR analysis

RNA isolation, cDNA synthesis and quantitative reverse transcription PCR analysis were performed as previously described by us [20]. The primer sequences used for CPNE1 mRNA detection were 5′-ACCCACTCTGCGTCCTT-3′ (forward) and 5′-TGGCGTCTTGTT GTCTATG-3′ (reverse), and the primers for miR-335-5p and U6 were purchased from RiboBio Co. Ltd. (Guangzhou, China). Ct values for CPNE1 mRNA and miR-335a-5p were equilibrated to those for *ACTB* mRNA and U6, respectively, which were used as internal controls. The △△Ct method was applied to determine the relative expression of these proteins.

### Generation of stable cell lines overexpressing CPNE1

To generate NSCLC cells in which CPNE1 is stably overexpressed, a 1626-bp fragment of the CPNE1 coding sequence was synthesized (Genewiz, Suzhou, China) and subcloned into a pLVX-IRES-Neo vector using the endonucleases *EcoR*I and *Xba*I for expression via a Lenti-X lentiviral expression system (Clontech, Mountain View, CA, USA). The CPNE1 expression construct was co-transfected with packaging plasmids into human embryonic kidney 293 T cells using Lipofectamine 2000 (Invitrogen). The empty vector served as a negative control. Human embryonic kidney 293 T cells were cultured in Dulbecco’s modified Eagle’s medium containing 10% FBS at 37 °C in a humidified 5% CO_2_ incubator for 48 h. After the incubation, the packaged lentiviruses were collected and used to infect A549 and H1299 cells. After 2 days, stable cells were selected with 400 μg/ml of G418 (Amresco, Solon, OH, USA).

### Western blot and co-immunoprecipitation assay

Western blot analysis was performed as previously described by us (Zhu et al., 2017). The antibodies used in the analysis were anti-CPNE1 (Z6), anti-pEGFR (Tyr1068) (1H12), anti-EGFR (A-10) (Santa Cruz, CA, USA), anti-pFAK (Tyr397) (D20B1), anti-FAK (D2R2E), anti-SRC (36D10), anti-pSRC (Tyr416), anti-pAKT (Ser473) (D9E), anti-AKT, anti-ERK (137F5), anti-pERK (Thr202/Tyr202) (D13.14.4E), anti-cyclinD1 (92G2), anti-MMP2 (D8N9Y), anti-MMP9 (603H) and anti-snail (C15D3) (Cell Signaling Technology, Danvers, MA, USA); anti-N-cadherin and anti-vimentin (RV202) (BD Biosciences, USA); and anti-β-actin and anti-mouse or anti-rabbit secondary antibodies (Cell Signaling Technology).

H1299-PLVX and H299-CPNE1 cells were cultured in a 10-cm plate until they reached 95−100% confluence. Then, the cells in each dish were washed twice with ice-cold PBS, collected by scraping, and lysed with 1 mL of modified RIPA buffer (Cell Signaling Technology, Danvers, MA, USA) containing a protease and phosphatase inhibitor cocktail (Sigma-Aldrich, St. Louis, MO, USA) for 30 min. Cell lysates were collected by centrifugation at 10,000 *g* at 4 °C for 15 min. Clear lysates were pre-cleared by addition of 25 μl of protein G bead slurry and incubated at 4 °C overnight with rotation. Supernatants were transferred to a new Eppendorf tube and incubated with 1 μg of rabbit anti-CPNE1 antibody with rotation overnight in a cold room; this was followed by additional incubation for 3−4 h with protein G beads. The beads were washed three times with RIPA buffer and then boiled in 2×SDS protein loading buffer for 5 min. Samples (20 μl) were loaded on SDS-PAGE gel for western blot analysis using the anti-CPNE1 antibody.

### Plasmid construction, transient transfection, and luciferase assay

To construct a plasmid containing the CPNE1 3′-untranslated region (3′-UTR) fused to the 3′-end of a luciferase reporter, a 225-bp fragment of the CPNE1 3′-UTR containing the miR-335-5p target sites (positions 137−143) predicted by TargetScan was chosen for the luciferase assay. The wild-type (psiCHECK2-CPNE1–3′-UTR) and one mutant fragment (psiCHECK2-CPNE1–3′-UTR-mutant) were directly synthesized (Genewiz, Suzhou, China) and fused to the 3′-end of a luciferase reporter (psiCHECK2 dual luciferase vector; Promega, Madison, WI, USA). A549 and H1299 cells were plated in a 24-well plate and cotransfected with the constructed plasmids with either miR-335-5p mimics or miR-negative control (miR-NC) using Lipofectamine 2000 (Life Technologies). The plates were maintained for 48 h, and then the cells were collected and luciferase activity was measured with the Dual-Luciferase Reporter Assay kit (Promega). Each experiment was conducted in triplicate.

### Cell proliferation analysis and drug treatment

Cell proliferation was examined using Cell Counting Kit-8 (Boster, Wuhan, China). Cells or the corresponding negative control cells were seeded in 96-well plates at a density of 2 × 10^3^ cells per well and further grown under normal culture conditions for 24, 48 and 72 h. Cell viability was determined according to the manufacturer’s instructions. The experiment was performed in triplicate. We also detected cell proliferation using a clonogenic assay. Briefly, cells transfected with miR-335-5p mimics and si-CPNE1 or si-NC were diluted in complete culture medium, and 200 cells were reseeded in a 60-mm plate. After incubation for 14–20 days, depending on cell growth rate, foci formed by least 50 cells were stained with Giemsa and counted. Cell viability was measured according to manufacturer’s instructions at several time points (24, 48 and 72 h). Each experiment was performed in triplicate. For drug treatment, stable CPNE1-knockdown cells were plated in 96-well plates, and gefitinib (gefitinib C#s1025; Selleck Chemicals, Houston, TX, USA) were added to the cultures. Cell viability was assessed at 72 h after the drug treatment.

### Wound healing, migration and invasion assays

Motility analysis of the cells was performed as previously described by us [20]. In brief, at 48 h after the transfection, the cells were seeded in 6-well plates until they formed a monolayer. Then, a scratch was made across the center of the well with the tip of a new 10-μl pipette tip, the culture was washed gently twice with PBS, and the medium was replaced with fresh medium. The cells were grown for an additional 24 h and were observed under a microscope (CKX41, Olympus). According to the instructions from the manufacturer, 5 × 10^4^ cells in a medium containing 1% FBS were seeded into the upper chamber of a Transwell insert, 800 μl of medium containing 10% FBS was placed into the lower chamber, and the entire chamber was incubated at 37 °C for 24 h. For the invasion assay, the inserts were coated with the Matrigel matrix (BD Science, Sparks, MD, USA) diluted in serum-free medium and incubated at 37 °C for 2 h. Finally, the cells were photographed and counted.

### Cell cycle analysis

According to the instructions of the Cell Cycle Analysis Kit (Beyotime, Shanghai, China), cells were cultured in 6-well plates and transfected with miR-NC, miR-335-5p, Si-NC or Si-CPNE1 for 72 h. The cells were then collected, washed with cold phosphate-buffered saline (PBS), fixed in 70% ethanol at 4 °C for 24 h, washed with cold PBS again and stained in a propidium iodide (PI)/RNase A mixture. Next, the cells were kept in the dark at 37 °C for 30 min and analyzed using a fluorescence-activated cell sorting (FACS) Caliber system (Beckman Coulter, Brea, CA, USA).

### Cell apoptosis analysis

Cells were transfected with miR-NC, miR-335-5p, Si-NC or Si-CPNE1. After 72 h, cells were harvested, washed, and resuspended in the binding buffer containing Annexin V/FITC and PI (Beyotime). The stained cells were then detected using the FACS Caliber system (Beckman Coulter).

### Animal experiments and immunocytochemistry staining

Female BALB/c athymic nude mice (4–6 weeks old and weighing 16–20 g) were purchased from the Experimental Animal Center of Soochow University and bred under pathogen-free conditions. All the animal experiments were carried out in accordance with the Guide for the Care and Use of Experimental Animals Center of Soochow University. To establish the lung carcinoma xenograft model, H1299 cells in which CPNE1 can be stably overexpressed were suspended in 100 ml of PBS and inoculated subcutaneously into the flanks of nude mice, which were randomly divided into two groups (8 mice in each group). Tumor volume (*V*) was determined by measuring the length *(L*) and width (*W*) with a vernier caliper and applying the following formula: *V* = (*L* × *W*^2^) × 0.5.

### Statistical analysis

Differences in CPNE1 and miR-335-5p expression between NSCLC tissues (T) and adjacent noncancerous lung tissues (N) were analyzed using a paired *t*-test (two-tailed). For cell lines, differences between two groups were assessed using an unpaired *t*-test (two-tailed). The clinicopathologic characteristics and expression levels of mRNA and miRNA in the NSCLC samples were compared using nonparametric tests (Mann-Whitney *U*-test for comparison between two groups, and the Kruskall-Wallis test for comparison between three or more groups). Two-way ANOVA was used to determine the difference in cell growth between two groups. Differences were considered to be significant at *P* < 0.05. All statistical analyses were performed using GraphPad Prism 5.02 (GraphPad, San Diego, CA, USA) and the SPSS 16.0 software (SPSS, Chicago, IL, USA).

## Results

### High incidence of CPNE1 overexpression in NSCLC tissues and cell lines

**Fig. 1:**
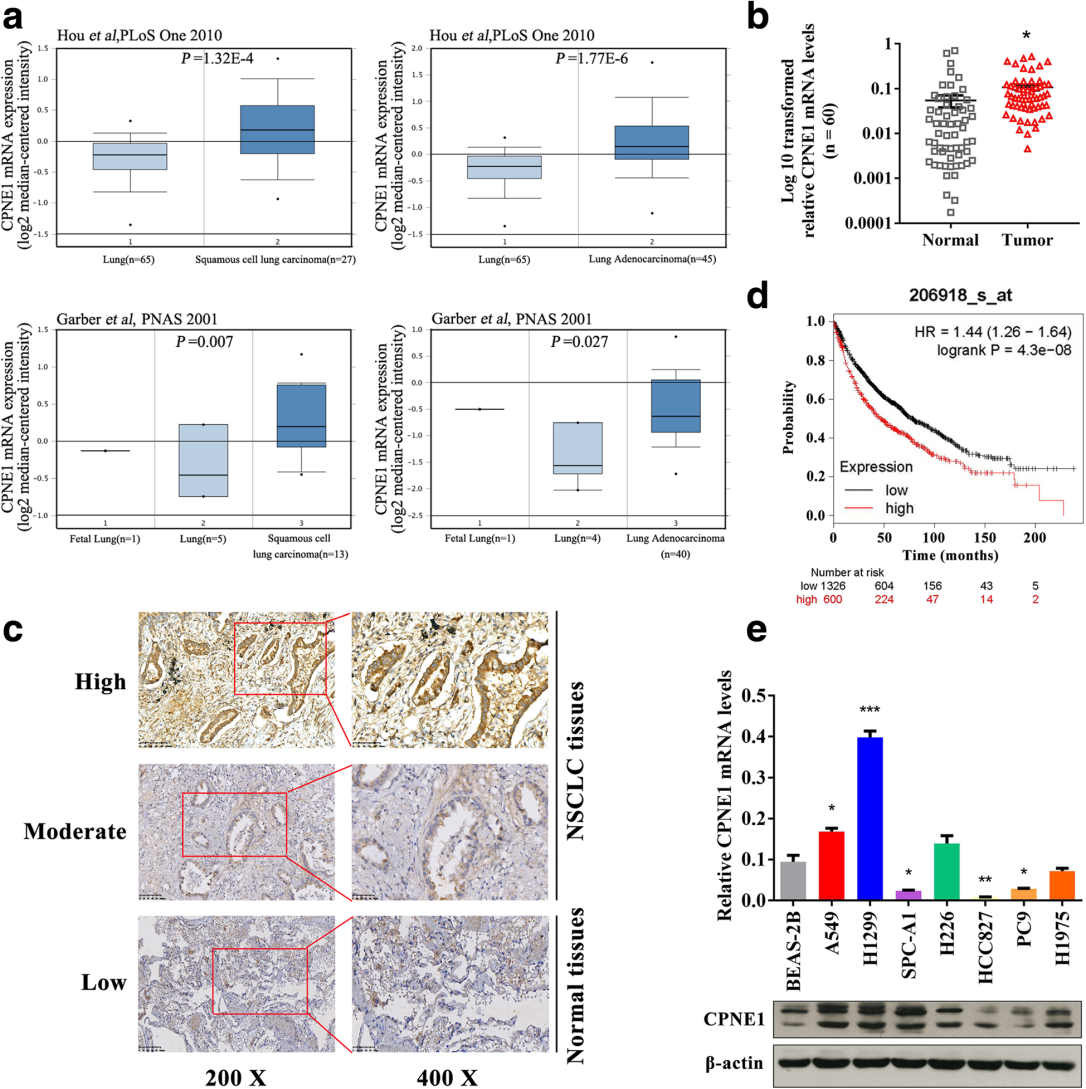
CPNE1 overexpression in NSCLC. **a** Data on CPNE1 mRNA expression in lung adenocarcinoma, squamous cell carcinoma and normal lung tissue from several study groups deposited in the Oncomine database (http://www.oncomine.org). **b** CPNE1 mRNA levels in 60 NSCLC tissues and paired noncancerous lung tissues. **c** Formalin-fixed and paraffin-embedded NSCLC tissues (*n* = 20) were subjected to IHC analyses of the CPNE1 protein. Representative images of CPNE1 antibody staining (high, moderate and low) in NSCLC tissues and normal tissues. **d** Effect of the CPNE1 expression level on overall survival in 1926 lung cancer patients; the Kaplan-Meier plots were generated using a Kaplan-Meier Plotter (http://www.kmplot.com). **e** The level of CPNE1 in human NSCLC cells was detected by qRT-PCR and western blot analysis. *P < 0.05; **P < 0.01; ***P < 0.001

Based on public data deposited in Oncomine (http://www.oncomine.org), we found that CPNE1 mRNA expression is significantly upregulated in lung adenocarcinoma and squamous cell carcinoma tissues compared to normal lung tissues (Fig. 1a). Further, detection of CPNE1 mRNA expression in 60 paired NSCLC tissues and adjacent noncancerous lung tissues showed that the CPNE1 mRNA levels were significantly higher in NSCLC tissues than in adjacent noncancerous lung tissues (Fig. 1b). No significant difference in the CPNE1 mRNA level was observed between NSCLC samples when they were classified according to age and gender; however, significant differences were observed according to lymph node metastasis, smoking habit of the patient and TNM stage (Table 1). Moreover, IHC analysis of the CPNE1 protein in 20 NSCLC samples showed that CPNE1 is mainly located in the cell membrane and cytoplasm of NSCLC cells (Fig. 1c). More importantly, it was found that upregulated expression of CPNE1 is significantly associated with poor survival, based on Kaplan-Meier analysis (Fig. 1d). In addition, qRT-PCR and western blot analysis of CPNE1 mRNA and protein expression, respectively, in six NSCLC cell lines and BEAS-2B cells (Fig. 1e) showed that the incidence of high CPNE1 expression was higher in the NSCLC tissues and cell lines than in the normal cell samples.

**Table 1:** Clinical characteristics and levels of miR-335-5p and *Copine1* mRNA expression in NSCLC tissues

Characteristics	*n* = 60(%)	miR-335-5p mRNA expression		*p* value	CPNE1 mRNA expression		*p* value
		high	low		high	low	
		(*n* = 15)	(*n* = 45)		(*n* = 53)	(*n* = 7)	
Age							
≤65	41 (68%)	9	32	0.432	36	5	0.851
> 65	19 (32%)	6	13		17	2	
Gender							
Male	38 (63%)	10	28	0.757	34	4	0.718
Female	22 (37%)	5	17		19	3	
Histology							
Adenocarcinoma	33 (55%)	7	26	0.479	30	3	0.661
Squamous cell carcinoma	17 (28%)	4	13		14	3	
Others	10 (17%)	4	6		9	1	
Smoking status							
Yes	33 (55%)	7	26	0.454	30	3	0.492
No	27 (45%)	8	19		23	4	
Clinical stage							
I/II	24 (40%)	4	20	0.224	21	3	0.870
III/IV	36 (60%)	11	25		32	4	
Lymph node metastasis							
No	24 (40%)	10	14	0.015	18	6	0.009
Yes	36 (60%)	5	31		35	1	

Date are presented as the mean ± SE values. Unpaired *t* test was used for comparison between two groups, and the Kruskal-Wallis test was for comparison of three or more groups

### Inhibition of in vitro cell growth, cell cycle progression and migration of NSCLC cells by CPNE1 knockdown

**Fig. 2:**
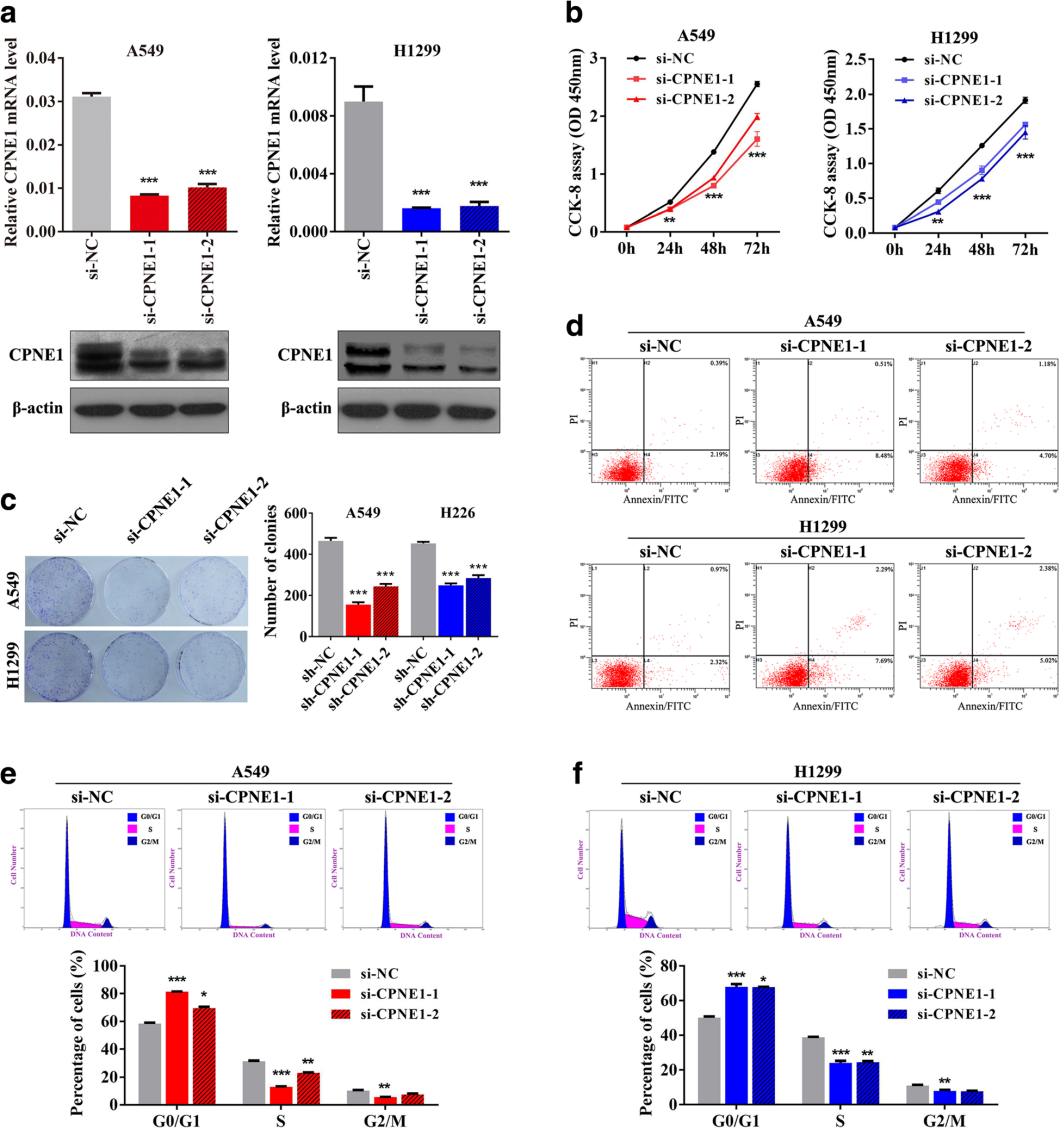
Inhibition of NSCLC cell proliferation and apoptosis by silencing of CPNE1. **a** CPNE1 mRNA and protein levels in CPNE1-silenced NSCLC cells. **b** CCK-8 assay of cell viability in NSCLC cell lines; cell viability was determined at 24, 48 and 72 h. **c** Representative images of the results of clonogenic analysis of cell proliferation in NSCLC cells. Bar charts showing clonogenic growth of cells. **d** Flow cytometry assay of A549 and H1299 cells (silenced for CPNE1 and compared with NC). Cells were harvested at 72 h after transfection and stained with Annexin V/FITC and propidium iodide (PI). **e**-**f** Flow cytometric analysis of NSCLC cell lines (CPNE1-silenced cells vs. NC cells). Cells were harvested at 72 h after transfection and stained with PI. The percentage of cells in each cell cycle phase is shown in the inset of each panel, in which the values represent the mean ± SD of three measurements. *P < 0.05; **P < 0.01; ***P < 0.001

The expression level of CPNE1 mRNA and protein was significantly reduced after transfection of A549 and H1299 cells with two small interfering RNAs (siRNAs) (Fig. 2a) against CPNE1. The CCK-8 assay showed that cell growth was significantly inhibited in cells with stable knockdown of CPNE1 compared with the control cells at 24 h, 48 h, and 72 h after transfection (Fig. 2b). We confirmed these findings by performing a clonogenic assay (Fig. 2c). The flow cytometry results indicated that transfection of NSCLC cells with si-CPNE1 resulted in an increase in apoptosis (Fig. 2d). Further, the proportion of cells in the G0/G1 phase was significantly higher and the proportion of cells in the S phase was significantly lower in the CPNE1-silenced cells than in the control cells (*P* < 0.05, Fig. 2e and f). These results indicate that CPNE1 can inhibit cell growth in NSCLC cells via its effects on cell cycle and apoptosis.

**Fig. 3:**
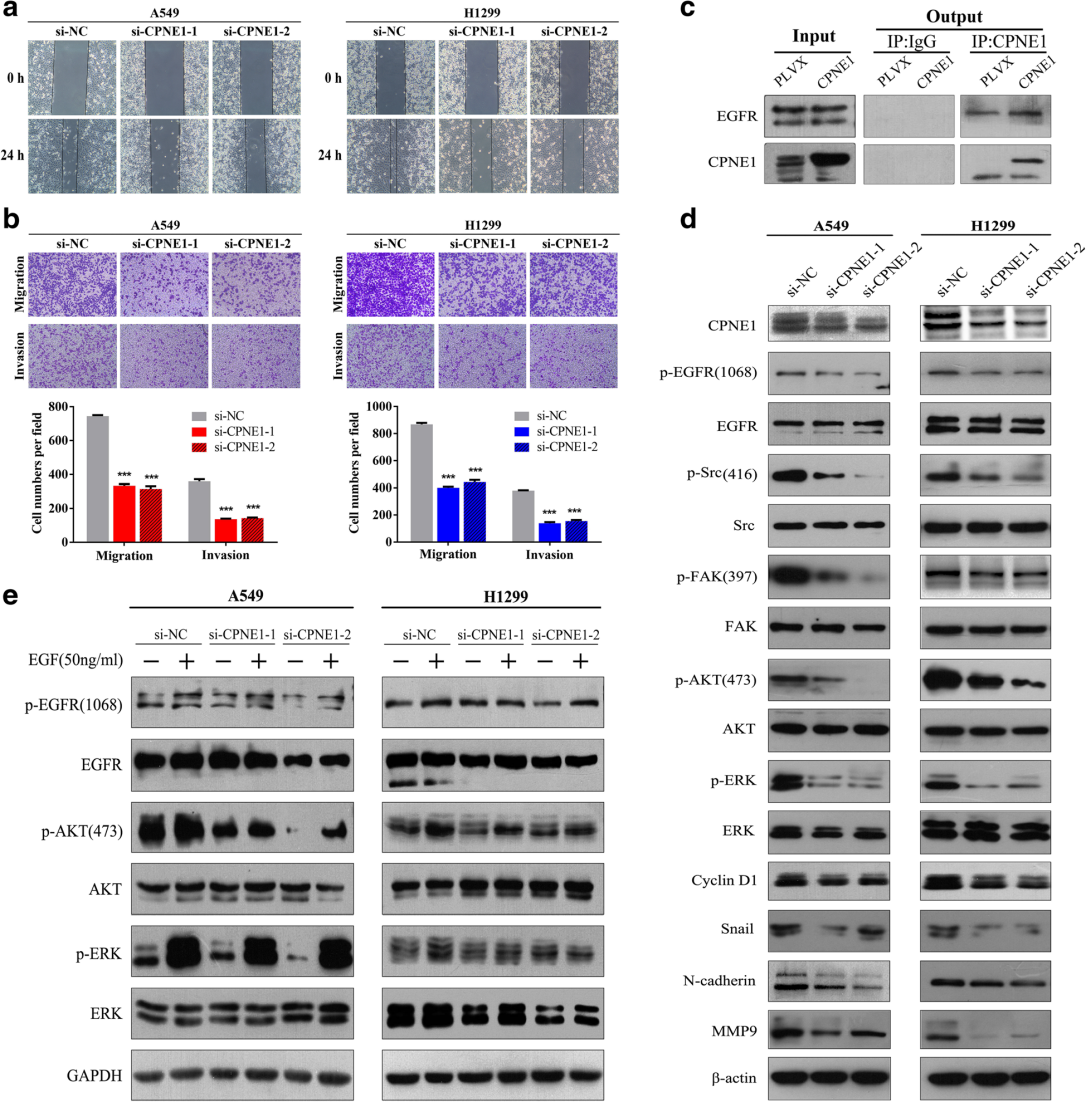
CPNE1 silencing-induced inhibition of the migratory and invasive abilities of NSCLC cells and their associated pathways. **a** The wound healing assay showed that the speed with which cells migrated towards the scratch was lower in CPNE1-silenced cells than in control cells. **b** CPNE1 silencing inhibited invasion and migration of NSCLC cells. CPNE1-silenced NSCLC cells were allowed to migrate through an 8-μM pore Transwell. The cells that migrated were stained and counted in at least three microscopic fields (magnification, × 100). Then, the cells were treated as above and allowed to invade through the Matrigel-coated membrane in Transwells. Invasive cells were stained and counted under a light microscope. **c** Co-immunoprecipitation of Copine-1 and EGFR. Copine-1 was immunoprecipitated from lysates of control and CPNE1-overexpressing H1299 cells using a specific monoclonal antibody. **d** p-EGFR and downstream signaling molecules were detected, and the data showed that the p-EGFR, p-Src, p-FAK, p-AKT and p-ERK levels were significantly decreased in the CPNE1-silenced cells compared with the control cells. **e** In the stable cell lines with CPNE1 knockdown, the EGF-induced increase in the level of p-EGFR and the other downstream signaling molecules levels was inhibited. *P < 0.05; **P < 0.01; ***P < 0.001

In the wound healing assay, the si-CPNE1-transfected NSCLC cells migrated towards the scratch more slowly than did the control cells (Fig. 3a). Transwell assay of the NSCLC stable cells lines further indicated that loss of CPNE1 considerably suppressed the migratory ability of NSCLC cells (Fig. 3b).

### Increase in in vitro cell growth and the migratory and invasive abilities of NSCLC cells by CPNE1 overexpression

To further investigate the function of CPNE1 in NSCLC cells, we first established A549 and H1299 cell lines with stable overexpression of CPNE1: this was confirmed by the high level of *CPNE1* mRNA and protein expression that was consistently observed in the cells overexpressing *CPNE1*. CCK-8 and clonogenic assays showed that the growth of cells overexpressing *CPNE1* was significantly promoted compared with the control cells. Moreover, wound healing and Transwell assays showed that overexpression of CPNE1 enhanced the migratory and invasive abilities of A549 and H1299 cells (Additional file 1: Figure S1). Collectively, these data strongly indicate that *CPNE1* acts as an oncogene in NSCLC progression.

### CPNE1 as a potential therapeutic target

Based on the findings reported in the literature [10, 11], we hypothesized that dysregulation of Copine 1 via the EGF signaling pathway and knockdown of CPNE1 expression could sensitize NSCLC cells to therapeutic agents. Firstly, we examined complexes of Copine 1 and EGFR using co-immunoprecipitation experiments. EGFR was found in immunoprecipitates of Copine 1 from lysates of H1299 cells with stable overexpression of CPNE1 (Fig. 3c). Then, we detected the molecular expression of p-EGFR and downstream signaling molecules. As illustrated in Fig. 3d, our data showed that the p-EGFR, p-Src, p-FAK, p-AKT and p-ERK levels were significantly decreased in the CPNE1-silenced cells compared with the control cells. Furthermore, in the cell lines with *CPNE1* knockdown, the EGF-induced increase in the level of p-EGFR and its downstream signaling molecules was inhibited (Fig. 3e).

Cells with stable CPNE1 overexpression were plated in 96-well plates, and then gefitinib was added to the cultures. Cell viability was assessed at 72 h after drug treatment. In the A549 and H1299 stable cell lines, the cells with CPNE1 overexpression were more resistant than the control cells when they were exposed to gefitinib. Furthermore, our western blotting data showed that in cells with CPNE1 overexpression, p-EGFR expression is increased and there is greater resistance to gefitinib (Additional file 2: Figure S2).

### Promotion of in vivo tumor growth by CPNE1 overexpression

**Fig. 4:**
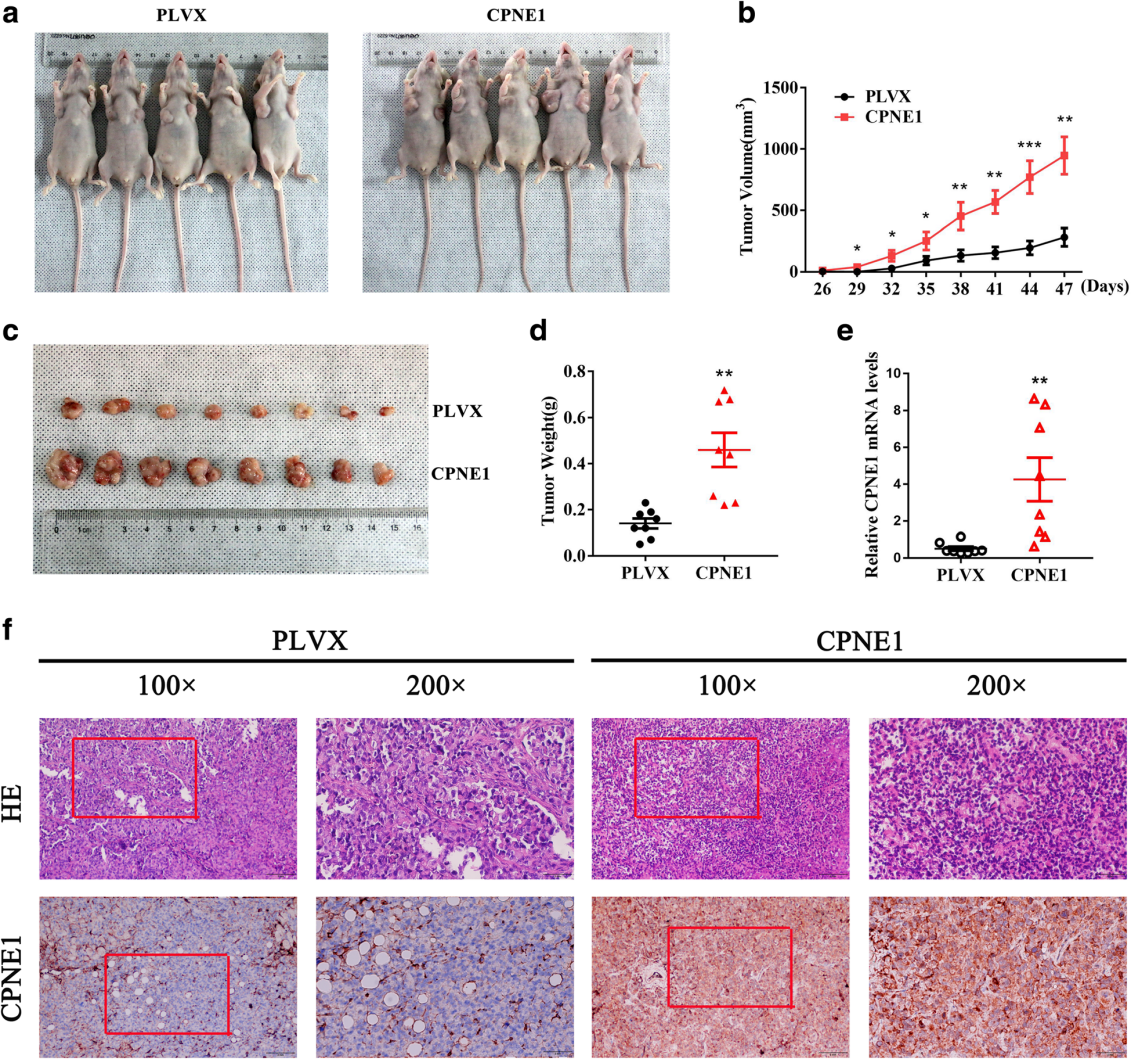
Promotion of in vivo tumor growth by CPNE1 overexpression. **a**-**c** CPNE1 overexpression in A549 cell xenografts in nude mice (*n* = 6) at the experimental endpoint; tumors were dissected and photographed as shown. Tumor growth curves in mice (*n* = 6 in each group) inoculated with the indicated cells at the indicated days **d** Each tumor formed was weighed. **e** CPNE1 mRNA expression in tumors was detected by qRT-PCR analysis. **f** Hematoxylin and eosin (H&E) staining confirmed the presence of tumor cells in the indicated tumor sections. Immunohistochemical staining for CPNE1 was quantified based on staining intensity. *P < 0.05; **P < 0.01; ***P < 0.001

To further assess the in vivo effect of CPNE1 overexpression on NSCLC cells, H1299 cells with stable CPNE1 overexpression were inoculated into BALB/C athymic mice. As shown in Fig. 4a−c, tumors formed by the cells with CPNE1 overexpression were much bigger in size than those formed from the control cells. In line with these results, tumor weight was found to be higher in cells with CPNE1 overexpression (Fig. 4d). The tissues resected from the xenograft tumors were analyzed to verify CPNE1 expression using qRT-PCR and IHC (Fig. 4e and f).

### CPNE1 is regulated by miR-335-5p and downregulation of miR-335-5p expression was detected in NSCLC tissues and cell lines

**Fig. 5:**
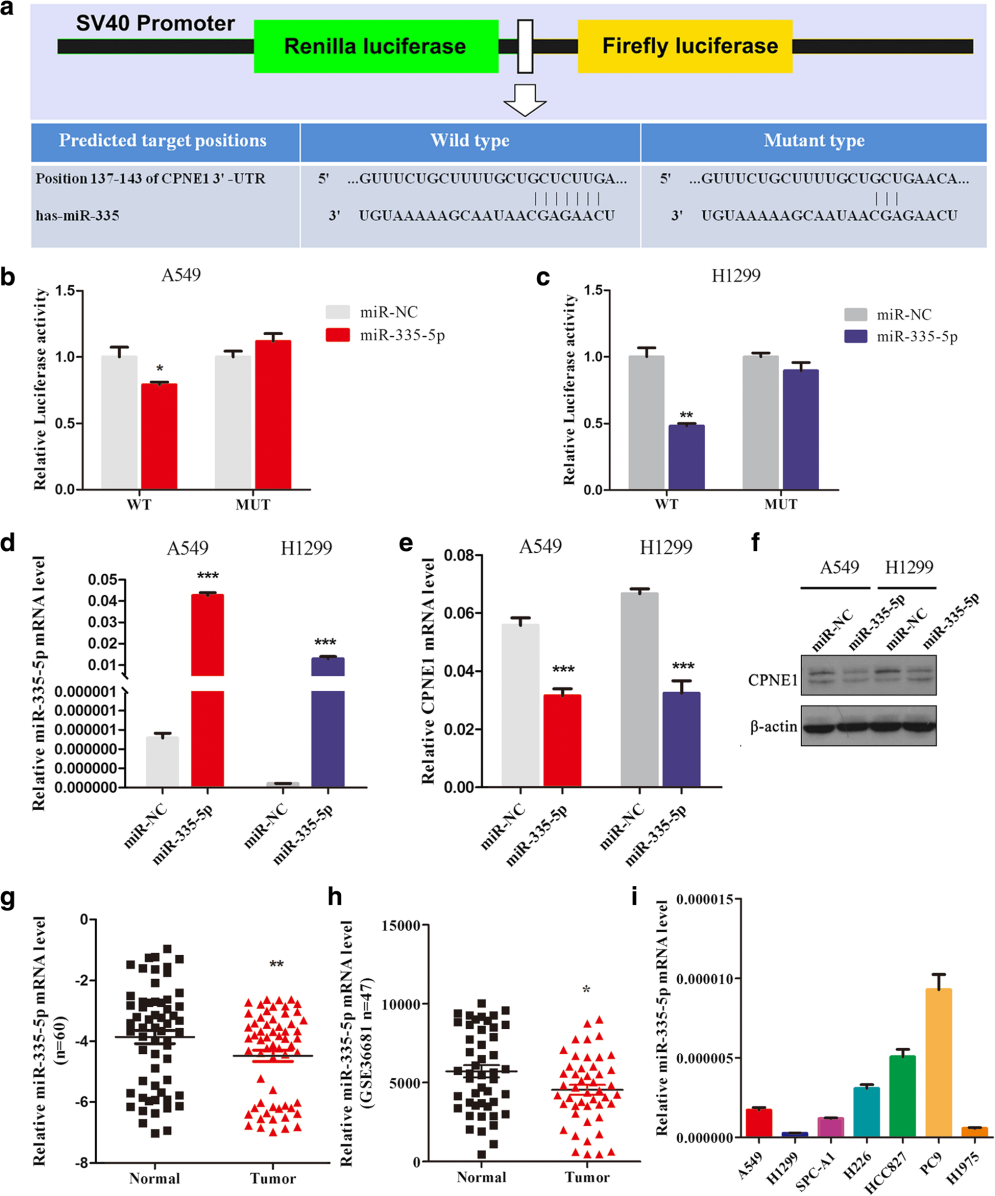
Regulation of CPNE1 expression by miR-335-5p and downregulation of miR-335-5p expression in NSCLC. **a** Schematic diagram showing the subcloning of the predicted miR-335-5p-binding site at positions 137–143 of the CPNE1 3′-UTR into a psiCHECK-2 luciferase construct. Predicted duplex formation between miR-335-5p and the wild-type or mutant miR-335-5p-binding site is indicated. **b**-**c** Luciferase activity of the construct containing the wild-type or mutant CPNE1 3′-UTR reporter gene in A549 and H1299 cells co-transfected with the negative control (NC) or miR-335-5p. Scrambled sequences were used as the NC. Relative Renilla luciferase activity was determined and normalized against firefly luciferase activity. **d**-**f** Expression of miR-335-5p and CPNE1 in NSCLC cells transfected with miR-335-5p mimics was detected by qRT-PCR and western blot analysis respectively. **g** Relative miR-335-5p levels in 60 NSCLC tissues (T) and paired noncancerous lung tissues (N). **h** A public Gene Expression Omnibus dataset (GSE36681) containing 47 NSCLC tissues and 47 normal lung tissues showed that miR-335-5p expression was downregulated in human NSCLC tissues. **i** qRT-PCR analysis of relative miR-335-5p expression in human NSCLC cell lines. *P < 0.05; **P < 0.01; ***P < 0.001

We used bioinformatics analysis to identify additional novel targets of miR-335-5p, and found that miR-335-5p could bind to the 3′-UTR of CPNE1 mRNA (TargetScanHuman: http://www.targetscan.org/). Therefore, it is possible that miR-335-5p inhibits CPNE1 expression by directly binding to its 3′-UTR region (Fig. 5a). To validate this prediction, the CPNE1 wild-type 3′-UTR (containing the miR-335-5p matching sequence) and CPNE1 MUT 3′-UTR (miR-335-5p mutated sequence) were constructed, and dual-luciferase reporter assay was performed in A549 and H1299 cells. The results showed that miR-335-5p significantly inhibited luciferase activity in cells transfected with the wild-type CPNE1 3′-UTR but did not repress luciferase activity in cells containing the mutant1&2 construct (Fig. 5b and c).

We also detected *CPNE1* expression in A549 and H1299 cells transfected with miR-335-5p, while the control cells were transfected with miR-NC. The data showed that the expression of miR-335-5p was increased in NSCLC cells transfected with the miR-335-5p mimics compared with the cells transfected with miR-NC (Fig. 5d). In correspondence with the expression of miR-335-5p, the level of CPNE1 was downregulated in cells transfected with the miR-335-5p mimics, as determined using qRT-PCR and western blotting analysis (Fig. 5e and f).

Our previous study using a microRNA array showed that miR-335-5p is expressed at a lower level in lung cancer tissues than in noncancerous tissues. Considering that dysregulation of miRNAs is associated with multiple biological processes, Gene Ontology analysis and pathway analysis were applied. Interestingly, miR-335-5p was predicted to play a significant role in the pathogenesis of lung cancer (Additional file 3: Figure S3).

To verify the results of the microRNA array, we detected the expression of miR-335-5p using qRT-PCR, and found that its expression was significantly reduced in tumor tissues compared with the paired noncancerous tissues from the same patient (Fig. 5g and Table 1). Furthermore, a public Gene Expression Omnibus dataset (GSE36681) containing 47 NSCLC tissues and 47 normal lung tissues showed that miR-335-5p expression was downregulated in human NSCLC tissues (Fig. 5h). Then, the expression of miR-335-5p was detected by qRT-PCR in seven NSCLC cell lines (Fig. 5i).

### Inhibition of NSCLC cell proliferation and migration by overexpression of miR-335-5p

**Fig. 6:**
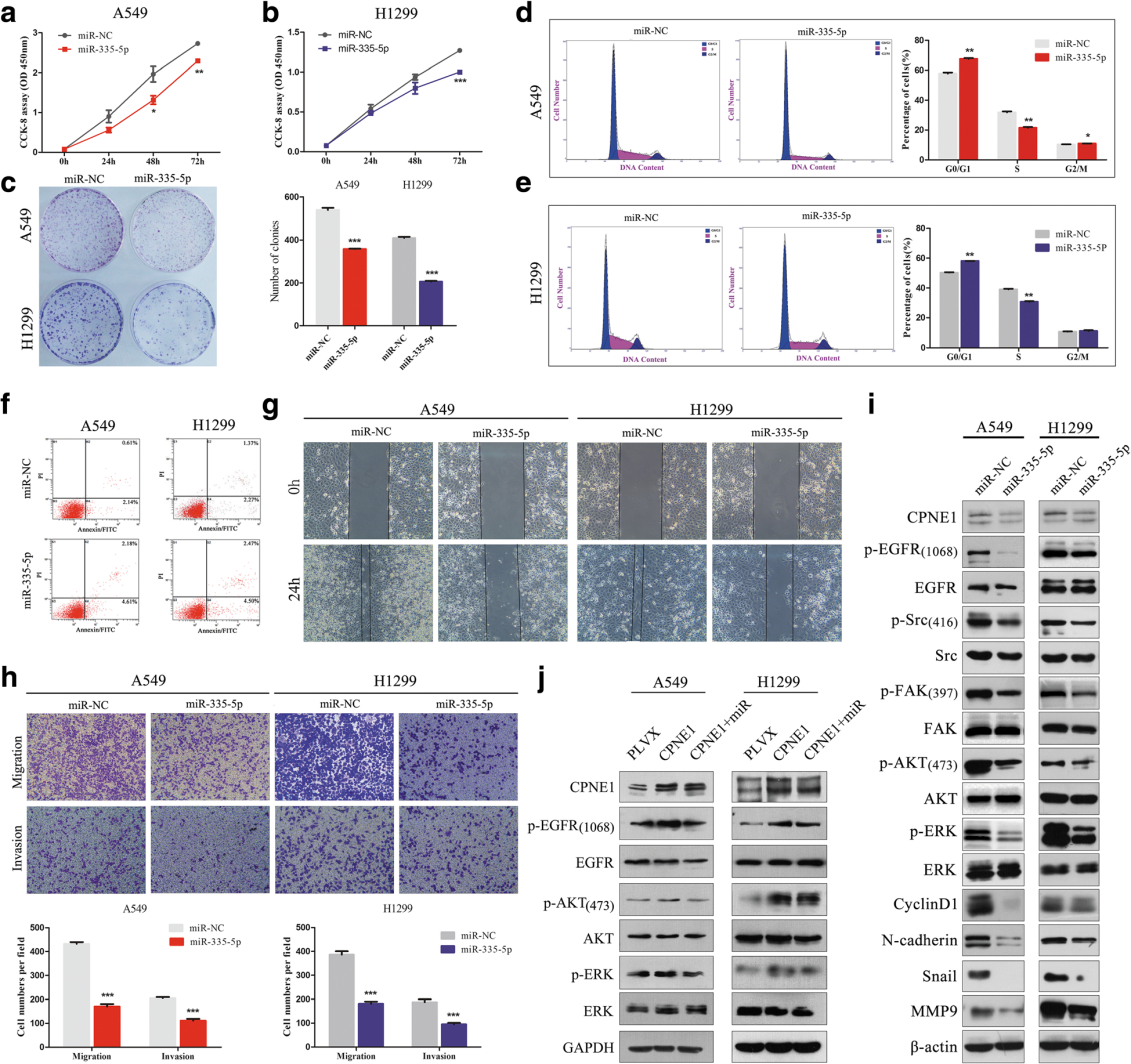
Inhibition of NSCLC cell proliferation and motility by overexpression of miR-335-5P. **a**-**b** CCK-8 assay of cell viability in NSCLC cell lines transfected with miR-335-5p mimics at 24, 48, and 72 h. **c** Representative images of the results of clonogenic analysis of cell proliferation in NSCLC cells. Bar charts showing the clonogenic growth of cells. **d** Flow cytometric analysis of the NSCLC cell lines (miR-335-5p vs. miR-NC cells). Cells were harvested at 72 h after transfection and stained with propidium iodide. The percentage of cells in each cell cycle phase is shown in the inset of each panel, in which the values represent the mean ± SD of three measurements. **e** A wound healing assay was performed to observe the role of miR-335-5p transfection in cells. The data showed that the speed with which the cells migrated towards the scratch was lower in cells transfected with the miR-335-5p mimics than in the control cells. **f** Flow cytometry assay of A549 and H1299 cells (cells transfected with miR-335-5p mimics vs. miR-NC). Cells were harvested at 72 h after transfection and stained with Annexin V/FITC and propidium iodide (PI). **g** Wound healing assay was performed to observe the role of cells transfected with the miR-335-5p mimics; the speed with which cells migrated towards the scratch was lower in the cells transfected with the miR-335-5p mimics than in the control cells. **h** Overexpression of miR-335-5p inhibits invasion and migration of NSCLC cells. The A549 and H1299 cell lines were transfected with miR-335-5p mimics and were allowed to migrate through 8-μM pore Transwells. The cells that migrated were stained and counted in at least three microscopic fields (magnification, × 100). Then, cells were treated as described before and allowed to invade through the Matrigel-coated membrane in the Transwells. The invasive cells were stained and counted under a light microscope. **i** The A549 and H1299 cells were treated with or without miR-30a-5p mimics for 72 h, respectively. The expression levels of p-EGFR, EGFR, p-Src, Src, p-FAK, FAK, p-AKT, AKT, p-ERK, ERK, EMT marker, snail and cyclin D1 were analyzed by western blotting. **j** Ectopic miR-335-5p led to lower p-EGFR expression in CPNE1-overexpressing cells than in the control cells. *P < 0.05; **P < 0.01; ***P < 0.001

To determine the function of miR-335-5p in NSCLC, we induced overexpression of miR-335-5p by using miR-335-5p mimics in NSCLC cells and studied their effects on cell growth. CCK-8 assays showed that NSCLC cells overexpressing miR-335-5p had significantly lower proliferation ability than the control cells (Fig. 6a and b). The results were confirmed by a clonogenic assay (Fig. 6c), the results of which indicated that miR-335-5p inhibits NSCLC cell proliferation.

To determine how miR-335-5p suppresses cell proliferation in NSCLC cells, we examined the distribution of cell cycle phases using flow cytometry and found that the proportion of cells in the G0/G1 phase was significantly higher and the proportion of cells in the S phase was significantly lower in lung cancer cells overexpressing miR-335-5p than in the control cells (*P* < 0.05, Fig. 6d and e). In addition, we found that transfection of NSCLC cells with miR-335-5p mimics had an effect on cell apoptosis (Fig. 6f, *P* < 0.001). These results indicated that miR-335-5p inhibits cell proliferation in NSCLC cells via its effects on cell cycle and apoptosis.

The wound healing assay was performed to observe the role of miR-335-5p transfection in A549 and H1299 cells. As shown in Fig. 6g, the speed with which cells migrated towards the scratch was lower in the cells transfected with the miR-335-5p mimics than in the control cells. Transwell assay of A549 and H1299 cells further indicated that loss of miR-335-5p considerably suppressed the migratory ability of NSCLC cells (Fig. 6h). Taken together, these observations indicate that miR-335-5p may have a tumor suppressor functions in NSCLC.

In line with the results for CPNE1-silenced cells, our data showed that the p-EGFR, p-Src, p-FAK, p-AKT and p-ERK levels were significantly lower in the miR-335-5p-overexpressing cell lines than in the control cells (Fig. 6i). In addition, we found that ectopic miR-335-5p resulted in lower p-EGFR expression in CPNE1-overexpressing cells than in the control cells (Fig. 6j).

## Discussion

The findings of the present study indicate that CPNE1 is a critical factor in the tumorigenesis of NSCLC and that its mechanism involves the EGFR signaling pathway. Additionally, CPNE1 was found to affect the efficacy of EGFR-targeted therapies in NSCLC cells with wild-type EGFR. Based on the present findings, future investigation into the molecular mechanisms of CPNE1-mediated drug resistance could help develop novel CPNE1-based therapeutic agents to improve the treatment of NSCLC.

The present study is the first to report that CPNE1 expression is upregulated and is positively correlated with the TNM stage and lymph node metastasis status in NSCLC patients; this finding is consistent with the results of a study on prostate cancer [12]. Furthermore, we demonstrated through CPNE1-silenced or CPNE1-overexpressing cells that CPNE1 is an oncogene that promotes in vitro cell growth and the migratory and invasive abilities of NSCLC cells; this finding is in line with the reported role of CPNE3 in NSCLC [9]. Moreover, CPNE1 was found to affect the efficacy of EGFR-targeted therapies in NSCLC cells with wild-type EGFR.

**Fig. 7:**
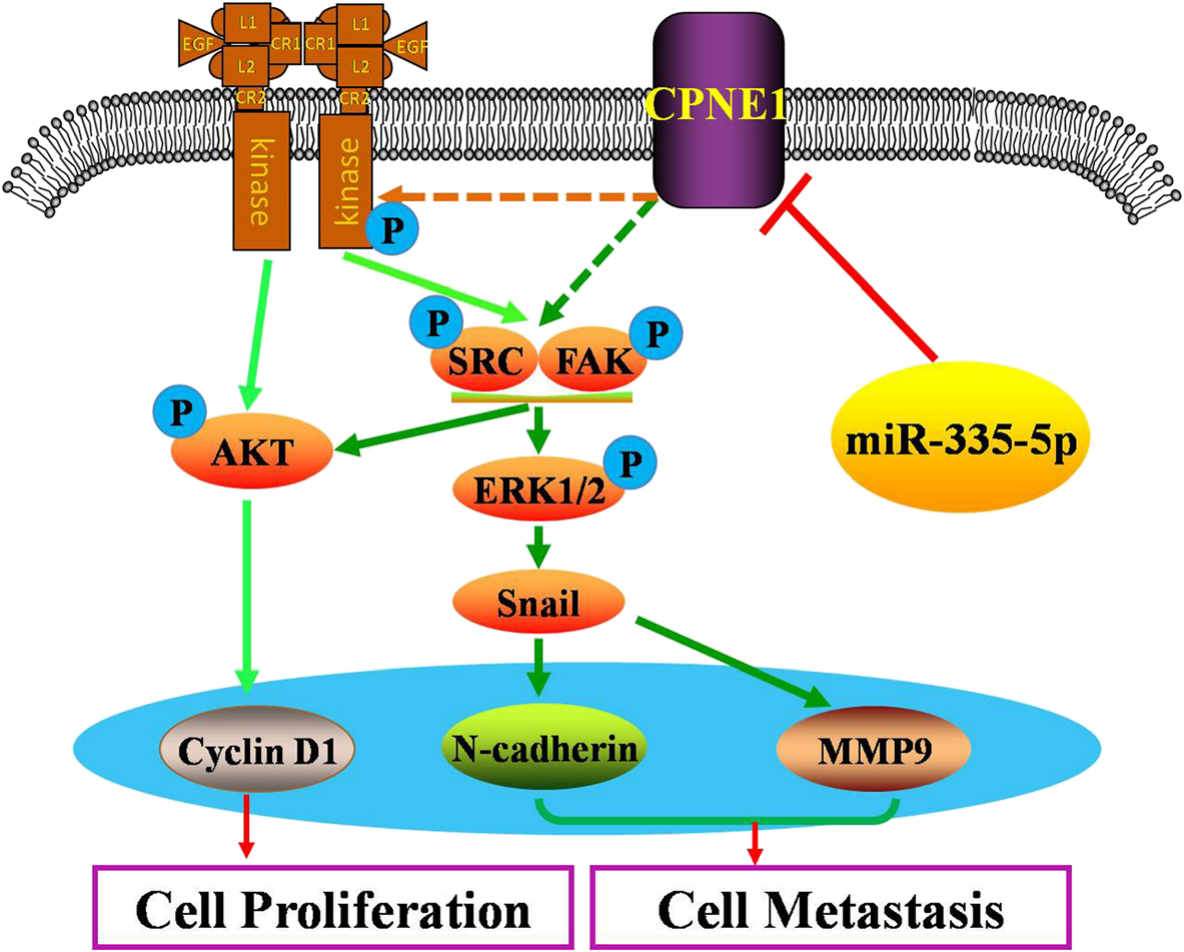
A working model of the mechanistic interaction of miR-335-5p and CPNE1 in the control of the EGFR signaling pathway: miR-335-5p regulates CPNE1 expression via EGFR signaling in NSCLC

Although the role of CPNE1 in cancer has been reported before, the underlying mechanisms are unclear. In the current study, we reveal that CPNE1 repression by miR-335-5p inhibits cell growth and metastasis in NSCLC cells via modulation of the EGFR signaling pathway, and establish the mechanistic interaction between CPNE1 and miR-335-5p in regulation of the lung cancer cell phenotype (Fig. 7).

miR-335-5p has been found to be one of the miRNAs that are downregulated in various types of solid tumors, including gastric cancer, renal cell cancer, hepatocellular cancer and pancreatic cancer [29–32]. Further, using gene and miRNA expression profiling in human high- and low-metastatic lung cancer cell strains, Chen et al. found that miR-335-5p might be one of the metastasis-related miRNAs in lung cancer [33]. These findings imply that miR-335-5p has some putative function in cancer, especially metastasis. However, the tumor suppressive role of miR-335-5p in NSCLC remains unclear. In the present study, we used bioinformatics analysis and found that the 3′-UTR of CPNE1 has a sequence that is complementary to that of miR-335-5p. Next, we used two methods to confirm that CPNE1 is a target of miR-335-5p. First, a dual-luciferase reporter assay was performed in A549 and H1299 cells, and it showed that miR-335-5p significantly inhibited luciferase activity in cells transfected with the wild-type CPNE1 3′-UTR but did not repress luciferase activity in cells containing the mutant construct. Second, ectopic expression of miR-335-5p in NSCLC cells was examined and found to be significantly increased, and CPNE1 mRNA and protein expression was found to have decreased. These findings indicate that both low miR-335-5p and high CPNE1 expression are found in NSCLC.

As mentioned in the literature review, the variation of microRNAs expression may act as a diagnostic and/or prognostic biomarker tool in human cancers, with different relationships in various cancers and their subtypes. Here, we need to pay more attention to that appreciation of molecular function of microRNAs in initiation and progression of cancer via targeting numerous tumour suppressor genes and oncogenes could help us to address a cancer therapeutic issue and open new avenues for gene therapy in those cancers where its tumor suppressive functions are dominant [20, 27]. We also need concern the points that a gene targeted by multiple miRNA or the multiple gene targeted by one miRNA [27, 34]. In the present study, although the evidence shows that dysregulated expression of CPNE1 is due to target binding of miR-335-5p in NSCLC, it is not surprising that other miRNAs may target CPNE1 and exert the same biological function in NSCLC or other human cancers.

Additionally, it has conclusively been shown the involvement of miRNA in acquired resistance to EGFR TKI. Li et al., have reported that miR-21 mediate acquired EGFR TKI resistance by targeting PTEN [35]. In addition, EGFR TKI combination with miRNA mimics or inhibitors has shown a synergistic effect in inhibiting NSCLC cell growth [20, 36]. Thus, it seems that miRNAs may represent promising candidates for adjuvant therapy for NSCLC patients who develop resistance to EGFR TKI treatment.

## Conclusions

In conclusion, our study is the first to report that CPNE1 expression is upregulated in NSCLC and correlates with a decrease in miR-335-5p expression. Furthermore, we found that miR-335-5p inhibits CPNE1 expression by directly targeting the CPNE1 3′-UTR, thereby repressing NSCLC cell proliferation and motility. Thus, our findings shed light on the mechanistic interaction between CPNE1 and miR-335-5p in NSCLC carcinogenesis. This miR-335-5p-mediated modulation of the EGFR signaling pathway via targeting of CPNE1 provides new insight into therapeutic strategies for NSCLC.

## Additional files

Additional file 1: Increase in NSCLC cell proliferation and motility by CPNE1 overexpression. (a) CPNE1 mRNA and protein levels in stable cell lines overexpressing CPNE1 (CPNE1-OE). (b) CCK-8 assay of cell viability in NSCLC cells; the results were detected at 24, 48 and 72 h. (c) Representative images of the results of clonogenic analysis of cell proliferation in CPNE1-OE and control cells. Bar charts showing the clonogenic growth of cells. (d) Wound healing assay was performed to observe the role of *CPNE1*-overexpressing cells; the speed with which cells migrated towards the scratch was higher in CPNE1-OE cells than in control cells. (e and f) CPNE1 overexpression promotes the invasion and migration of NSCLC cells. CPNE1-overexpressing NSCLC cells were allowed to migrate through an 8-μM pore Transwell. The cells that migrated were stained and counted in at least three microscopic fields. Then, the cells were treated as described above and allowed to invade through the Matrigel-coated membrane in Transwells. Invasive cells were stained and counted under a light microscope. Values shown are the mean ± SE values from three measurements. **P < 0.01; ***P < 0.001. (TIF 5351 kb)

Additional file 2: CPNE1 overexpression can decrease the sensitivity of NSCLC cells to therapeutic agents. On dysregulation of CPNE1 via the EGFR signaling pathway, the molecular expression of EGFR and its downstream signaling molecules were detected. (a) CPNE1 overexpression can decrease the sensitivity of NSCLC cells to therapeutic agents. (b) On dysregulation of CPNE1 via the EGFR signaling pathway, the molecular expression of EGFR and its downstream signaling molecules were detected. *P < 0.05; **P < 0.01; ***P < 0.001. (TIF 2702 kb)

Additional file 3: Decrease in miR-335-5p expression in NSCLC tissues and cell lines and the associated with biological processes and signaling pathways. (a) miR-335-5p expression was significantly decreased in NSCLC. Fold change > 2 or < 0.5 and ii) False discovery rate (FDR) < 0.05 and *P* < 0.005. Each row represents individual miRNAs, and the columns represent tumor and normal tissue samples. The color scale depicts the relative expression ratio of an miRNA following normalization (red, high expression level; green, low expres sion level). (b) Gene ontology and pathway analyses. (TIF 1787 kb)

## Abbreviations

CPNE1: Copine 1
IHC: Immunohistochemical
microRNA: miRNA
NSCLC: Non-small cell lung cancer
qRT-PCR: Quantitative reverse transcription–PCR
shRNAs: Short hairpin RNAs
