# Supplementary figures and images for: CPNE1 is a target of miR-335-5p and plays an important role in the pathogenesis of non-small cell lung cancer
Source: J Exp Clin Cancer Res. 2018 Jul 3;37:131. doi: 10.1186/s13046-018-0811-6 (PMC6029376; doi:10.1186/s13046-018-0811-6)

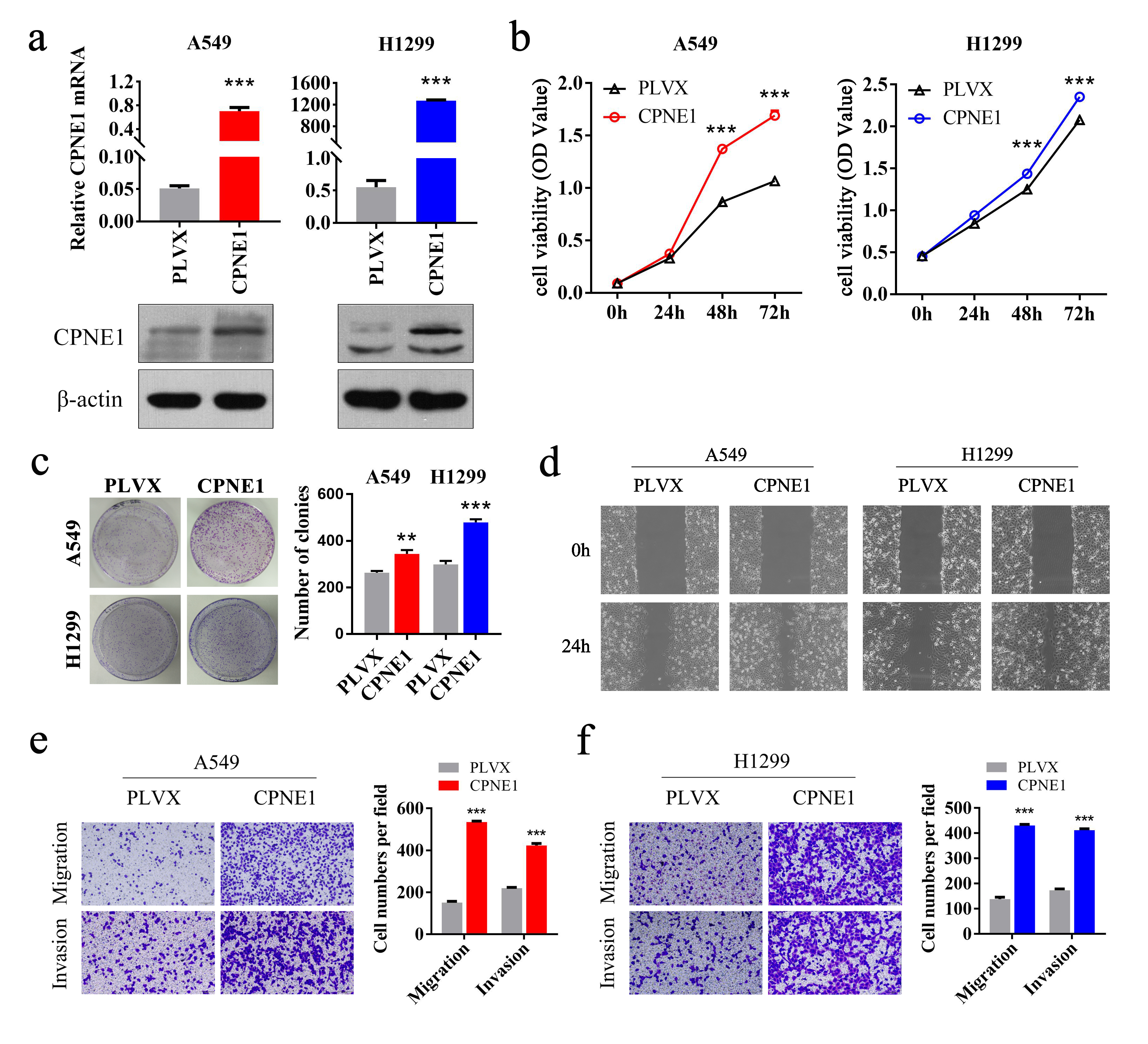

Supplement: Supplementary file 1 — Increase in NSCLC cell proliferation and motility by CPNE1 overexpression. (a) CPNE1 mRNA and protein levels in stable cell lines overexpressing CPNE1 (CPNE1-OE). (b) CCK-8 assay of cell viability in NSCLC cells; the results were detected at 24, 48 and 72 h. (c) Representative images of the results of clonogenic analysis of cell proliferation in CPNE1-OE and control cells. Bar charts showing the clonogenic growth of cells. (d) Wound healing assay was performed to observe the role of CPNE1-overexpressing cells; the speed with which cells migrated towards the scratch was higher in CPNE1-OE cells than in control cells. (e and f) CPNE1 overexpression promotes the invasion and migration of NSCLC cells. CPNE1-overexpressing NSCLC cells were allowed to migrate through an 8-μM pore Transwell. The cells that migrated were stained and counted in at least three microscopic fields. Then, the cells were treated as described above and allowed to invade through the Matrigel-coated membrane in Transwells. Invasive cells were stained and counted under a light microscope. Values shown are the mean ± SE values from three measurements. **P < 0.01; ***P < 0.001. (TIF 5351 kb) [file 13046_2018_811_MOESM1_ESM.tif]

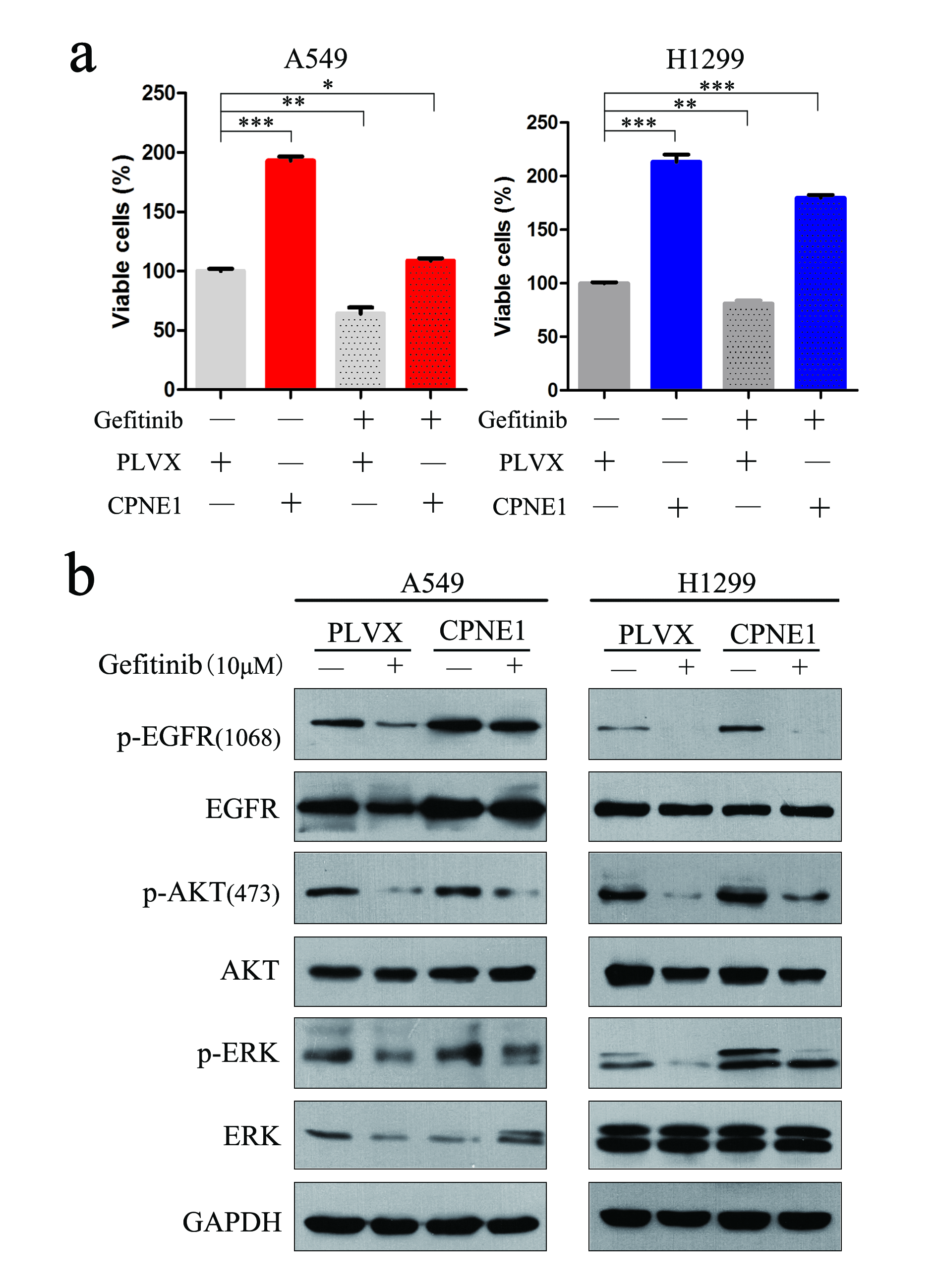

Supplement: Supplementary file 2 — CPNE1 overexpression can decrease the sensitivity of NSCLC cells to therapeutic agents. On dysregulation of CPNE1 via the EGFR signaling pathway, the molecular expression of EGFR and its downstream signaling molecules were detected. (a) CPNE1 overexpression can decrease the sensitivity of NSCLC cells to therapeutic agents. (b) On dysregulation of CPNE1 via the EGFR signaling pathway, the molecular expression of EGFR and its downstream signaling molecules were detected. *P < 0.05; **P < 0.01; ***P < 0.001. (TIF 2702 kb) [file 13046_2018_811_MOESM2_ESM.tif]

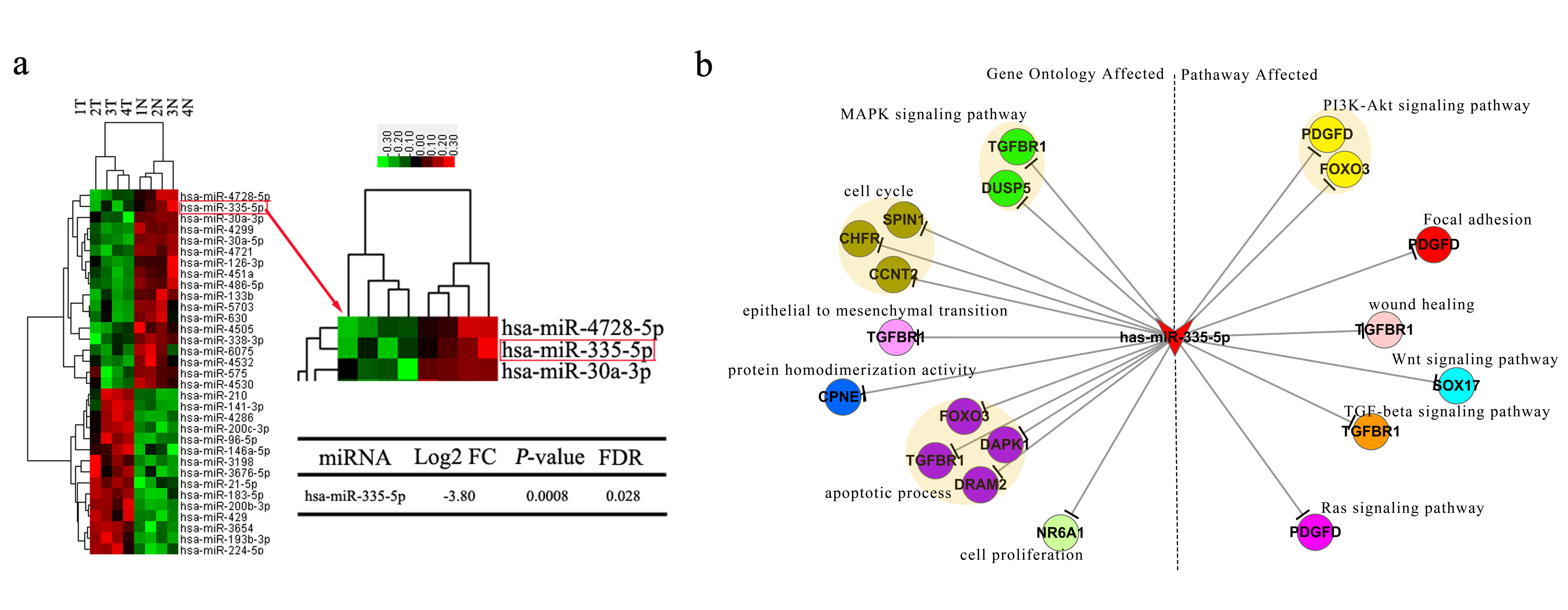

Supplement: Supplementary file 3 — Decrease in miR-335-5p expression in NSCLC tissues and cell lines and the associated with biological processes and signaling pathways. (a) miR-335-5p expression was significantly decreased in NSCLC. Fold change > 2 or < 0.5 and ii) False discovery rate (FDR) < 0.05 and P < 0.005. Each row represents individual miRNAs, and the columns represent tumor and normal tissue samples. The color scale depicts the relative expression ratio of an miRNA following normalization (red, high expression level; green, low expres sion level). (b) Gene ontology and pathway analyses. (TIF 1787 kb) [file 13046_2018_811_MOESM3_ESM.tif]
